# Comparative retrieval analysis of a novel anatomic tibial tray backside: alterations in tibial component design and surface coating can increase cement adhesions and surface roughness

**DOI:** 10.1186/s12891-022-05446-y

**Published:** 2022-05-19

**Authors:** Dominic T. Mathis, Joshua Schmidli, Felix Amsler, Johann Henckel, Harry Hothi, Alister Hart, Michael T. Hirschmann

**Affiliations:** 1 University of Basel, Basel, Switzerland; 2 Department of Orthopaedic Surgery and Traumatology, Kantonsspital Baselland, Bruderholz, Switzerland; 3 Amsler Consulting, Basel, Switzerland; 4 Royal National Orthopaedic Hospital, Stanmore, England UK

**Keywords:** Total knee arthroplasty, Tibial tray, Retrieval analysis, Implant-cement interface, Surface roughness, TKA revision reason, Cement adhesions

## Abstract

**Background:**

With the Persona® knee system a novel anatomic total knee design was developed, which has no pre-coating, whereas the predecessor knee system is pre-coated with polymethylmethacrylate (PMMA). Joint registry data have shown no decrease in risk of aseptic revision of PMMA pre-coated tibial components compared with non-pre-coated implants. The aim of this retrieval study was to compare the amount of cement adhesions, geometry and surface features between the two knee designs and to correlate them with the underlying reason for revision surgery.

**Methods:**

Retrieval analysis was performed of 15 NexGen® and 8 Persona® fixed-bearing knee implants from the same manufacturer retrieved from two knee revision centres. A photogrammetric method was used to grade the amount of cement attached to the tibial tray backside. The geometry and dimensions of the tibial trays, tray projections and peripheral lips were measured using digital callipers and compared between the two different designs. To measure the surface roughness on the backside of the tibial tray, a contact profilometer was used*.* To investigate differences between the two designs statistical analyses (t-test) were performed.

**Results:**

All Persona® trays showed evidence of cement adhesion with a % area of 75.4%; half of the NexGen® trays had cement adhesions, with a mean value of 20%. There was a significant difference in the percentage of area covered by cement between the two designs (*p <* 0.001). Results from the contact profilometer revealed that Persona® and NexGen® tray backsides showed a similar lateral (1.36 μm and 1.10 μm) and medial (1.39 μm and 1.12 μm) mean surface roughness with significant differentiation (*p <* 0.05) of the lateral and medial roughness values between the two designs. Persona® stems showed a significantly higher mean surface roughness (1.26) compared to NexGen® stems (0.89; *p <* 0.05).

**Conclusion:**

The novel anatomic knee system showed significantly more cements adhesions and a higher surface roughness which was most likely attributed to the most obvious design and coating alteration of the tibial tray. This study provides first retrieval findings of a novel TKA design recently introduced to the market.

## Introduction

Aseptic loosening represents the overall most common reason for revision in both cemented and uncemented total knee arthroplasty (TKA) and accounts for a third of all failures [1–11]. Retrieval studies have demonstrated that one of the causes of aseptic loosening is the osteoclastic-mediated bone resorption stimulated by polyethylene (PE) wear particles [12]. However, along with improved PE quality PE wear, subsequent osteolysis and loosening have become less frequent [7, 13–15]. Stress shielding, which is significantly influenced by the design and material of the tibial tray [16–19], stem length, and geometry [11, 20, 21] can be cited as another explanation. Furthermore, there is scientific evidence that both the cement type (high viscosity) and the application methods may have a relevant influence on aseptic tibial loosening caused by debonding of the tibial implant from the cement interface [22–24].

A novel total knee system, introduced to the European market in 2012, was developed to improve the mechanics of the knee replacement by making a more anatomically accurate knee implant [25]. More anatomical implant designs may optimize clinical outcome with an improved fit to patient anatomy [26]. In a recent study, Mathis et al. have demonstrated that PE damages of the novel anatomic knee system are not significantly different from those of its predecessor knee system [27]. The only difference they were able to show was a significantly smoother surface of the articulating side of the tibial tray compared to the predecessor knee system [27]. However, the study did not reveal retrieval results regarding the tibial tray backsides of the two implant designs. This is relevant because the two implants differ not only in terms of their design philosophy but also regarding their surface coatings. The novel anatomic knee system has no pre-coating whereas the predecessor is generally pre-coated with polymethylmethacrylate (PMMA) [28]. In a large contemporary joint registry with information collected on more than 16′000 predecessor cemented total knee fixed-bearing tibial tray implants, pre-coating the tibial component did not decrease the risk of aseptic revision compared with non-pre-coated implants [29]. The influence of the geometrics of tibial keels on aseptic loosening rates has been sparsely studied [21, 30, 31]. There is evidence that keels with a rectangular cross-section are more efficient than those with a triangular cross-section; short-keeled cemented tibial components showed an increased risk for aseptic loosening [21, 30].

To justify the use of the novel knee system over its well-established predecessor, post-market surveillance by means of retrieval data are vital as emphasized by the new Medical Device Regulation (MDR) [32]. Therefore, the primary objective of this retrieval study was to compare the amount of cement adhesion between the two knee designs from the same manufacturer. Secondly, the geometry and surface features of the implants were analyzed and compared and correlated with the underlying reason for revision surgery. It was hypothesized that no significant differences between the two designs are shown.

## Material and methods

### Retrieval cohort

This study examined all Persona® (*n =* 8) and NexGen® (*n =* 15) TKA implants consecutively received at our centre since 2016; all are produced by a single manufacturer (Zimmer Biomet, Warsaw, Indiana, USA). All tibial trays including stem were cemented during primary implantation. The implants were removed either in a specialized knee revision centre in Finland or in Switzerland by one fellowship trained senior knee surgeon in each centre. Both surgeons used the same technique with an oscillating saw and chisels during the tibial tray removal. All implants received at the retrieval centre were included in the study with the exception of implants revised due to infection (limited comparability).

TKA specifications and patient demographics for each case are presented in Table 1.

**Table 1:** Patient demographics. SD, standard deviation; OA, osteoarthritis; PS, posterior stabilized; CR, cruciate retaining

Case number	Gender	Age, yrs	Time to revision, yrs	Reason(s) for revision	Design, Type	Revision surgeon
1	F	51.3	0.4	Patellofemoral, stiffness, malalignment	NexGen®, PS	Surgeon 1
2	M	50.9	3.0	Instability	Persona®, PS	Surgeon 1
3	F	71.1	1.0	Instability, patellofemoral	Persona®, CR	Surgeon 1
4	F	76.6	9.0	Instability	NexGen®, PS	Surgeon 2
5	F	61.8	5.9	Periprosthetic fracture	NexGen®, CR	Surgeon 2
6	F	71.3	5.9	Instability	NexGen®, CR	Surgeon 2
7	F	67.3	7.8	Malalignment	NexGen®, CR	Surgeon 2
8	M	70.6	1.6	Instability	Persona®, PS	Surgeon 1
9	F	67.8	3.5	Instability, stiffness	Persona®, CR	Surgeon 1
10	F	69.2	1.4	Instability, patellofemoral	Persona®, PS	Surgeon 1
11	F	66.3	14.8	Progression of OA	NexGen®, PS	Surgeon 2
12	F	72.2	1.5	Instability, patellofemoral	Persona®, PS	Surgeon 1
13	F	52.4	0.9	Stiffness	NexGen®, CR	Surgeon 2
14	F	69.3	1.9	Instability	NexGen®, PS	Surgeon 2
15	F	79.6	9.7	Instability	NexGen®, CR	Surgeon 2
16	M	69.3	5.8	Instability	NexGen®, CR	Surgeon 2
17	F	84.2	10.1	Instability	NexGen®, CR	Surgeon 2
18	F	79.8	14.1	Instability	NexGen®, CR	Surgeon 2
19	F	72.8	13.1	Instability	NexGen®, CR	Surgeon 2
20	F	80.2	1.2	Stiffness	NexGen®, CR	Surgeon 2
21	M	70.5	3.2	Instability, malalignment	Persona®, CR	Surgeon 1
22	F	58.3	18.1	Instability	NexGen®, PS	Surgeon 2
23	F	66.0	2.2	Instability	Persona®, CR	Surgeon 1

The novel anatomic implants (Persona®) comprised a cruciate-retaining (CR, *n =* 4) and posterior-stabilized design (PS, *n =* 4); the tibial tray was made from wrought Tivanium® (Ti-6Al-4 V) alloy. All eight Persona® tibial trays had a fixed bearing (FB) configuration. All tibial inserts were made of vitamin-E doped and highly cross-linked polyethylene with antioxidant protection*.* These implants were retrieved from five (62.5%) female and three (37.5%) male patients, with a mean (standard deviation, SD) age of 67.3 (± 6.91) years and a mean (SD) time to revision of 2.2 (± 0.94) years. The main reason for revision was instability (*n =* 8, 100%; Table 2).

**Table 2:** Patient demographics by implant type. SD, standard deviation; OA, osteoarthritis; PS, posterior stabilized; CR, cruciate retaining; the percentages totalled > 100% because some knees had more than one reason for revision recorded

Design, type	Gender (F:M)	Age at revision, mean and SD (yrs)	Time to revision, mean and SD (yrs)	Reason(s) for revision
Persona®, total	5:3	67.3 (± 6.9)	2.2 (± 0.94)	Instability (*n =* 8, 100%); patellofemoral problem (*n =* 3, 37.5%); malalignment (*n =* 1, 12.5%); stiffness (*n =* 1, 12.5%)
Persona®, CR	3:1	68.9 (± 2.4)	2.5 (± 1.1)	Instability (*n =* 4, 100%); patellofemoral problem (*n =* 1, 25%); malalignment (*n =* 1, 25%); stiffness (*n =* 1, 25%)
Persona®, PS	2:2	65.7 (± 10)	1.9 (0.7)	Instability (*n =* 4, 100%); patellofemoral problem (*n =* 2, 50%)
NexGen®, total	14:1	69.4 (± 10.1)	7.9 (± 5.5)	Instability (*n =* 9, 60%); stiffness (*n =* 3, 20%); malalignment (*n =* 2, 13.3%); others (periprosthetic fracture, progression OA, *n =* 2, 13.3%); patellofemoral problem (*n =* 1, 6.6%)
NexGen®, CR	9:1	71.9 (± 9.7)	7.4 (± 4.4)	Instability (*n =* 6, 60%); stiffness (*n =* 2, 20%); malalignment (*n =* 1, 10%); others (periprosthetic fracture *n =* 1, 10%)
NexGen®, PS	5:0	64.4 (± 9.8)	8.9 (± 7.7)	Instability (*n =* 3, 60%); stiffness (*n =* 1, 20%); malalignment (*n =* 1, 20%); others (progression OA *n =* 1, 20%); patellofemoral problem (*n =* 1, 20%)

The predecessor implants (NexGen®) consisted of two different designs: CR-Flex (*n =* 10), and Legacy® posterior-stabilized (LPS)-Flex (*n =* 5), all with a FB design, the tibial tray was made from Ti-6Al-4 V alloy. Unlike Persona®, the vast majority of the NexGen® tibial backside and stem finishes are pre-coated with a thin layer of PMMA, less than 20% are non-pre-coated [28]. The tibial inserts were all made of Prolong® highly crosslinked ultrahigh-molecular-weight polyethylene (UHMWPE). These implants were retrieved from 14 (93%) female and one (7%) male patients, with a mean (SD) age of 69.4 (±10.1) years. The main reason for revision was also instability (*n =* 9, 60%) and the mean (SD) time to revision was 7.9 (±5.5) years; the latter was significantly longer for the predecessor compared to the novel knee system (*p <* 0.01; Tables 2 and 3).

**Table 3:** Mean values and standard deviations (SD) of surface roughness values and hood scores of all implants investigated; N of all revision reasons. Comparison between the two implant designs using R^2^ *P*-values < 0.05 were considered statistically significant

Implant type	Total (*N =* 23)	NexGen® (*N =* 15)	Persona® (*N =* 8)	Comparison		Comparison – corrected for time to revision	
Cement adhesions	Mean, SD	Mean, SD	Mean, SD	R^2^	P	R^2^	P
Tibial tray backside, %	0.39 +/−0.32	0.20 +/− 0.21	0.75 +/− 0.08	0.70	.000	0.59	.000
Dimensions	Mean, SD	Mean, SD	Mean, SD	R^2^	P	R^2^	P
Stem, mm	39.6 +/−4.6	41.2 +/−4.44	36.5 +/−3.4	0.24	.016	0.13	.093
Tray Lip, mm	0.57 +/− 0.03	0.57 +/− 0.03	0.56 +/− 0.04	0.02	.533	0.09	.199
Tray thickness, mm (including lip)	3.81 +/− 0.6	3.62 +/− 0.05	4.2 +/−1.00	0.21	.033	0.14	.090
Surface roughness (Ra)	Mean, SD	Mean, SD	Mean, SD	R^2^	P	R^2^	P
Tibial back lateral Ra	1.19 +/− 0.3	1.10 +/− 0.24	1.36 +/− 0.35	0.18	.047	0.19	.044
Tibial back medial Ra	1.21 +/− 0.32	1.12 +/− 0.28	1.39 +/− 0.34	0.16	.050	0.13	.098
Tibial stem Ra	1.02 +/− 0.34	0.89 +/− 0.19	1.26 +/− 0.44	0.27	.011	0.17	.055
Tibial back & stem Ra	1.14 +/− 0.3	1.04 +/− 0.20	1.34 +/− 0.37	0.23	.021	0.18	.046
Time to revision	5.92 +/−5.23	7.92 +/−5.5	2.2 +/− 0.94	0.29	0.009	n.a.	n.a.
Reason for revision	N (%)	N (%)	N (%)	R^2^	P	R^2^	P
Instability	17 (74)	9 (60)	8 (100)	0.19	.039	0.32	.006
Malalignment	3 (13)	2 (13)	1 (13)	0.00	.957	0.01	.607
Patellofemoral	4 (17)	1 (7)	3 (38)	0.15	.068	0.04	.360
Stiffness	4 (17)	3 (20)	1 (3)	0.00	.669	0.16	.070
Others (periprosthetic fracture, progression OA)	2 (10)	2 (13)	0 (0)	0.05	.301	0.01	.657

### Sample preparation

All components were decontaminated using 10% formaldehyde solution (Solmedia Ltd., UK), followed by rinsing with water. The tibial tray backside and stem surfaces were prepared by using methylated spirit 99% (Solmedia Ltd., UK) to gently remove biomaterial without affecting cement adhesion.

### Study design

**Fig. 1:**
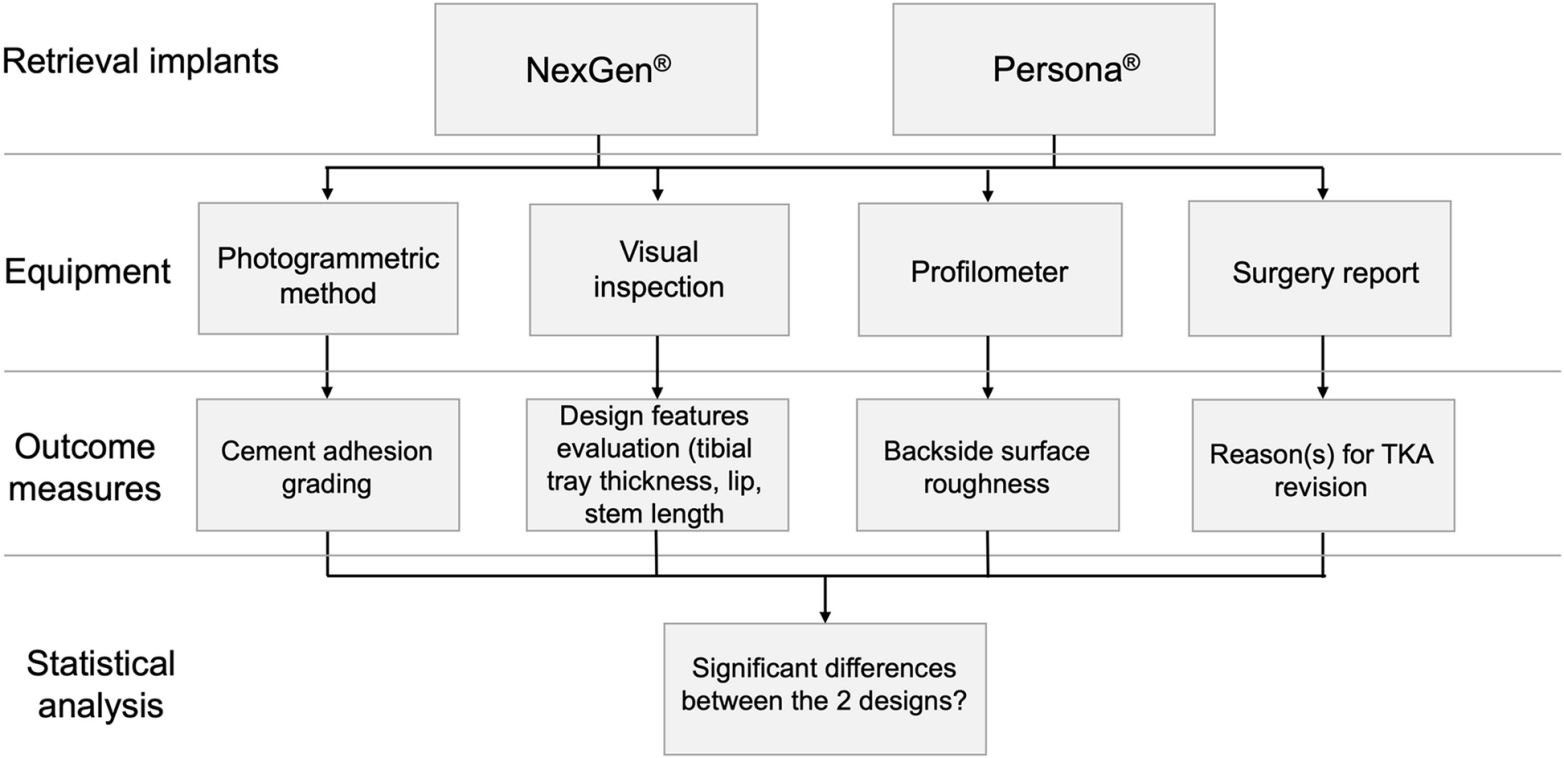
Flow chart showing the study design; TKA, total knee arthroplasty

The following retrieval analyses of the tibial tray backside were performed: (1) macroscopic analysis of cement adhesions, using a published photogrammetric grading method, (2) assessment of geometry and dimensions, (3) surface roughness measurement and (4) compared findings between the two knee designs. In addition, reasons for TKA revision were collected from surgery reports (Fig. 1).

### Grading of tibial tray backside cement adhesion

**Fig. 2:**
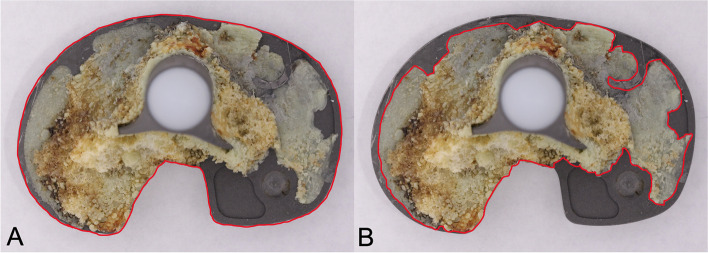
Example of sample analysed using the photogrammetric method [33]. **A** Total tibial tray backside surface contours highlighted in red. **B** Amount of surface covered by cement highlighted in red

A published photogrammetric method [33, 34] was used to grade the amount of cement attached to the tibial tray backside. High-resolution images of the tibial tray backside were captured using an EOS 5D Mark II camera (Canon Inc., Tokyo, Japan) (Fig. 2). The images were analyzed using public domain software (ImageJ 1.4.3.6.7, Broken Symmetry Software). First, the area covered by cement was measured and subsequently divided by the total backside area, in order to obtain the percentage of the area of interest.

### Design features assessment

**Fig. 3:**
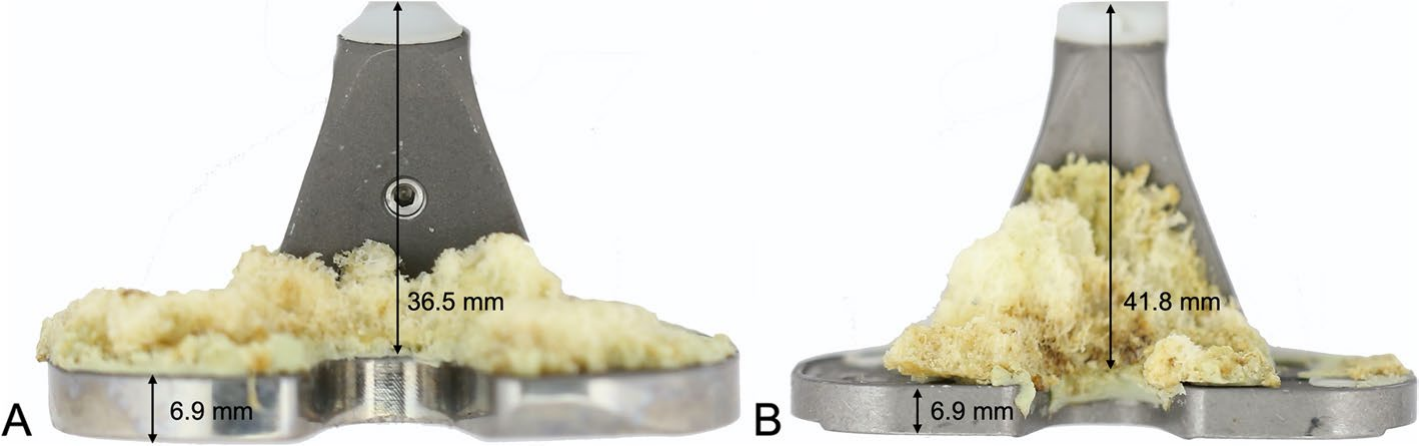
Design features analyzed by visual inspection: tibial tray thickness (A, B, 6.9 mm) and stem length (A, 36.5 mm; B. 41.8 mm). **A**, novel anatomic knee system (Persona®); **B**, predecessor knee system (NexGen®)

The geometry and dimensions of the tibial trays, tray projections (stem and/or fins) and peripheral lips were measured using digital callipers (Digimatic Absolute AOS; Mitutoyo, Kawasaki, Japan) and compared between the two different designs. Figure 3 shows the design features that were analyzed.

### Articulating surface roughness of metal components (profilometer)

To measure the surface roughness on the backside of the tibial tray, a well-established method was applied by using a contact profilometer Talyrond 365 (Taylor Hobson, Leicester, UK) with a 5-μm probe [27]. Surface roughness is defined as the average of the absolute values of the surface height deviations measured from the mean plane. The implant was positioned on the spindle, and measurements were taken using a 5 μm contact stylus. Six vertical traces were acquired on both the backside and stem of tibial trays, for a total of 12 traces for each implant. Measurements were performed while avoiding areas surface-damaged by scratches made during the revision surgery. Mean values for each design iteration were calculated (Table 3).

### Statistical analysis

Data was analysed using SPSS for Windows, version 26.0 (Armonk, NY: IBM Corp, USA) by an independent professional statistician.

A post hoc analysis using G*Power (version 3.1.9; University of Kiel, Germany) tested for correlations, that, for the given *N =* 23, an effect size rho = 0.53 can be found with a power of 80% with a two-sided p of 0.05.

All statistical tests were two-tailed. A *p*-value < 0.05 was considered significant. To compare mean values, t-tests for independent samples for group differences were used (e.g. comparison of the two component types). Pearson correlations were calculated for interval data and phi coefficients to compare binary variables.

To exclude a possible influence of time to revision on the measured variables in this study, the differences between implant types were corrected for time to revision for all parameters investigated using partialized values.

## Results

### Grading of tibial tray backside cement adhesion

**Fig. 4:**
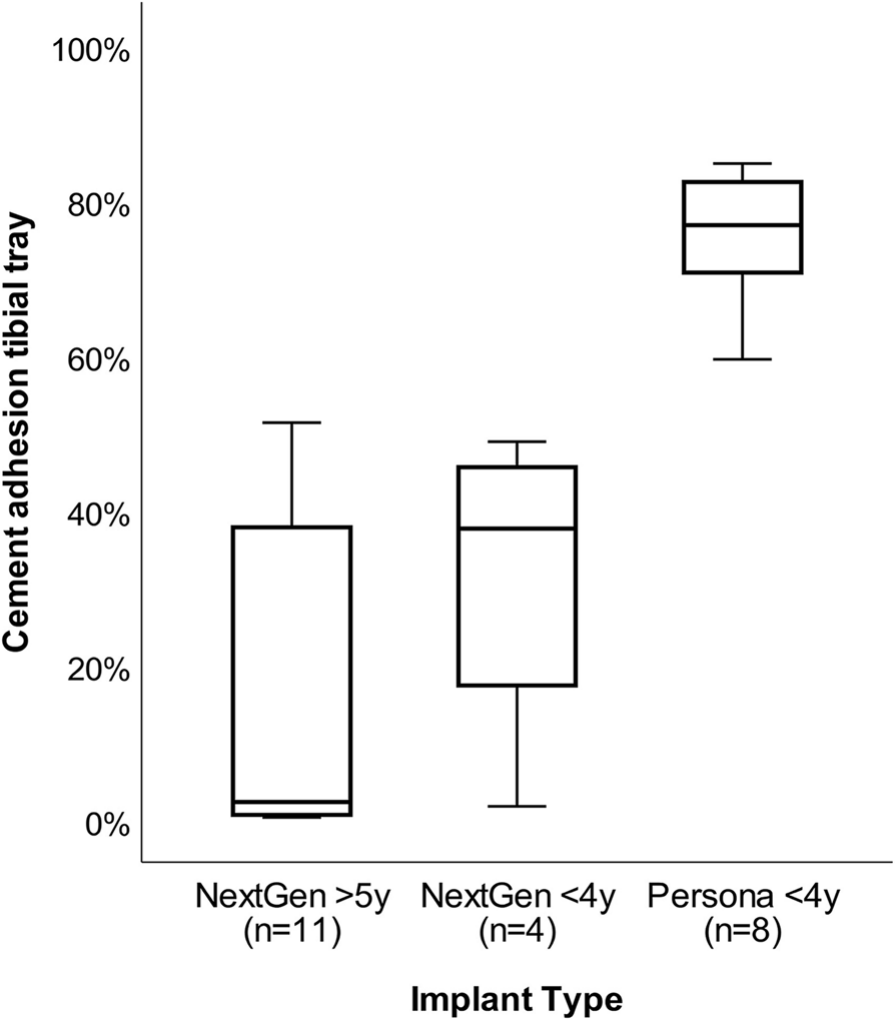
Box plots showing comparison of area covered by cement in the two designs analyzed. The difference between the novel anatomic knee system (Persona®) and the predecessor knee system (NexGen®) was significant (*p <* 0.001). The partitioning of NexGen® according to time to revision shows that time to revision did not influence the amount of cement adhesions significantly

**Fig. 5:**
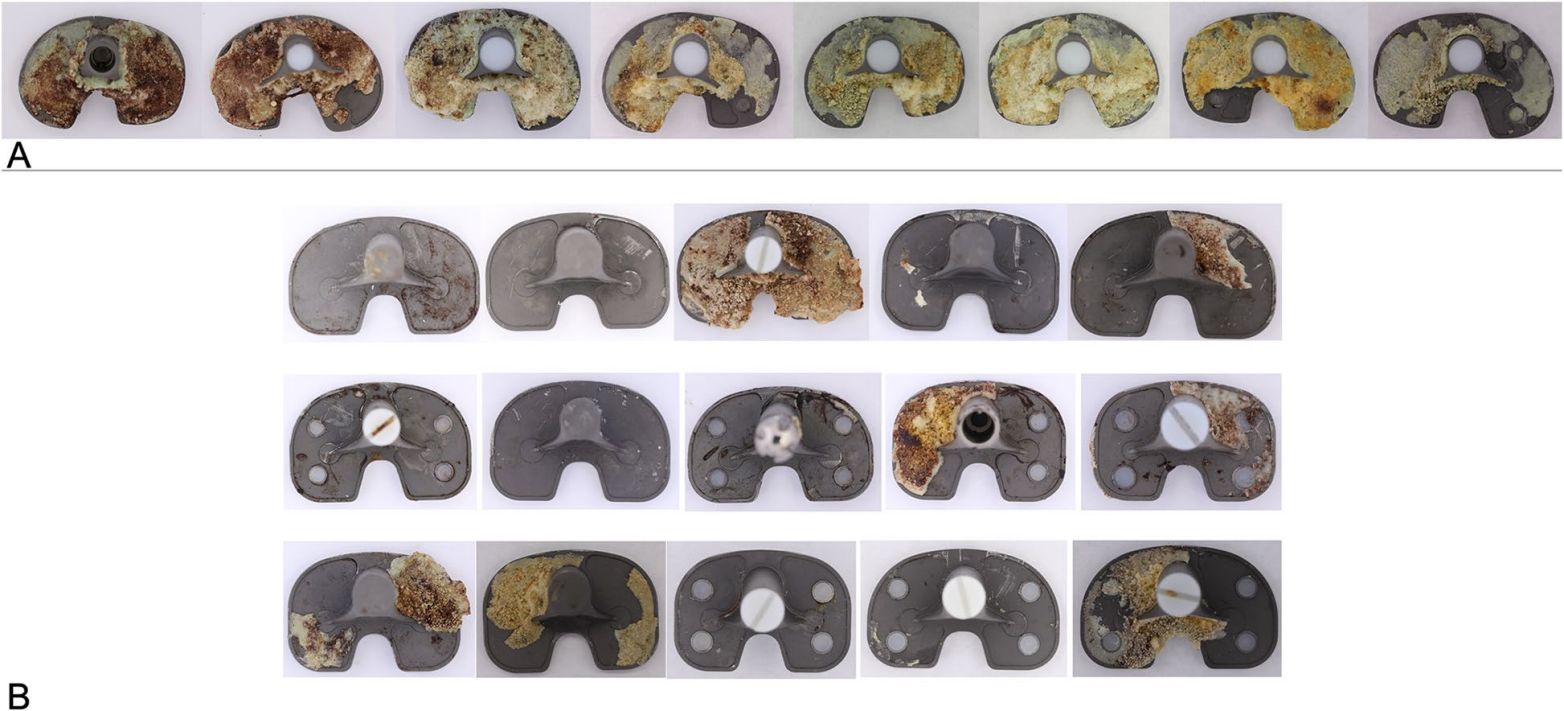
Picture showing the entire cohort, divided by design: **A**) novel anatomic knee system (Persona®), **B**) predecessor knee system (NexGen®)

The percentage of tibial tray backside covered by cement was highly variable. Figure 4 illustrates the distribution of percentage of area covered by cement in the two designs. It clearly appears that NexGen® implants revised in the same period as Persona® implants (< 4 years) showed a very similar distribution of cement adhesions compared to the ones revised between 4 and 18 years. All Persona® trays (*n =* 8, 100%) showed evidence of cement adhesion with a mean % area of 75.4% (range 59.4 to 84.7%). Half of the NexGen® trays (*n =* 7, 47%) had cement adhesions with a range of 32.9 to 51.2%; the other half (*n =* 8, 53%) showed nearly no cement adhesions with a range of 0.2 to 3.0% and an overall mean value of 20%. Overall, there was a significant difference in the percentage of area covered by cement between the two designs (*p <* 0.001). Figure 5 shows images of all of the components examined.

Between Persona® and NexGen® design there was a significant difference in the time to revision (*p <* 0.05); the mean values (±SD) were 2.2 (±0.9) and 7.9 (±5.5) years, respectively (Table 3). With regard to the demographic data collected, there was a increase in younger-aged (mean ± SD 61.5 ± 10.2) females (93%) in the NexGen® group, while the Persona® prosthesis was implanted more in older patients (mean ± SD 65.1 ± 7.4) with a higher proportion of males (38%). At the time of revision, however, the Persona® group showed a lower age (mean ± SD 67.3 ± 6.9) compared to the NexGen® group (mean ± SD 69.4 ± 10.1). None of the differences shown were significant.

### Visual inspection

Visual inspection revealed substantial differences between Persona® and NexGen® designs.

Overall, NexGen® implants had the thinner tray dimensions (mean ± SD = 3.62 ± 0.05 mm including the lip of the tray) compared to Persona® (mean ± SD = 4.2 ± 1 mm). The difference between the two designs was significant (*p* < 0.05).

Considering cemented tibial tray projections, Persona® and NexGen® showed a straight, linear, and central stem, with two diagonal/triangular fins, and mean (± SD) lengths of 36.5 ± 3.4 mm and 41.2 ± 4.4 mm, respectively. The difference in length between the Persona® and NexGen® stem was significant (*p* < 0.05).

Regarding peripheral lips, the Persona® trays showed a mean (± SD) depth of 0.56 ± 0.04 mm, while NexGen® designs showed mean values of 0.57 ± 0.03 mm; this difference was not significant. Table 3 summarizes the measurements taken, showing mean and SD values.

### Articulating surface roughness of metal components (profilometer)

Results from the contact profilometer revealed that Persona® and NexGen® tray backsides showed a similar lateral (1.36 ± 0.35 μm and 1.10 ± 0.24 μm; *p <* 0.05) and medial (1.39 ± 0.34 μm and 1.12 ± 0.28 μm; *p* = 0.05) mean (±SD) surface roughness with significant differentiation (*p <* 0.05) of the lateral and medial roughness values between the two designs (Table 3). Medial and lateral roughness did not differ and were within 1 SD for 22 of the 23 components, only in 1 component (4%) the lateral roughness value was increased by more than one SD compared to the medial one.

**Fig. 6:**
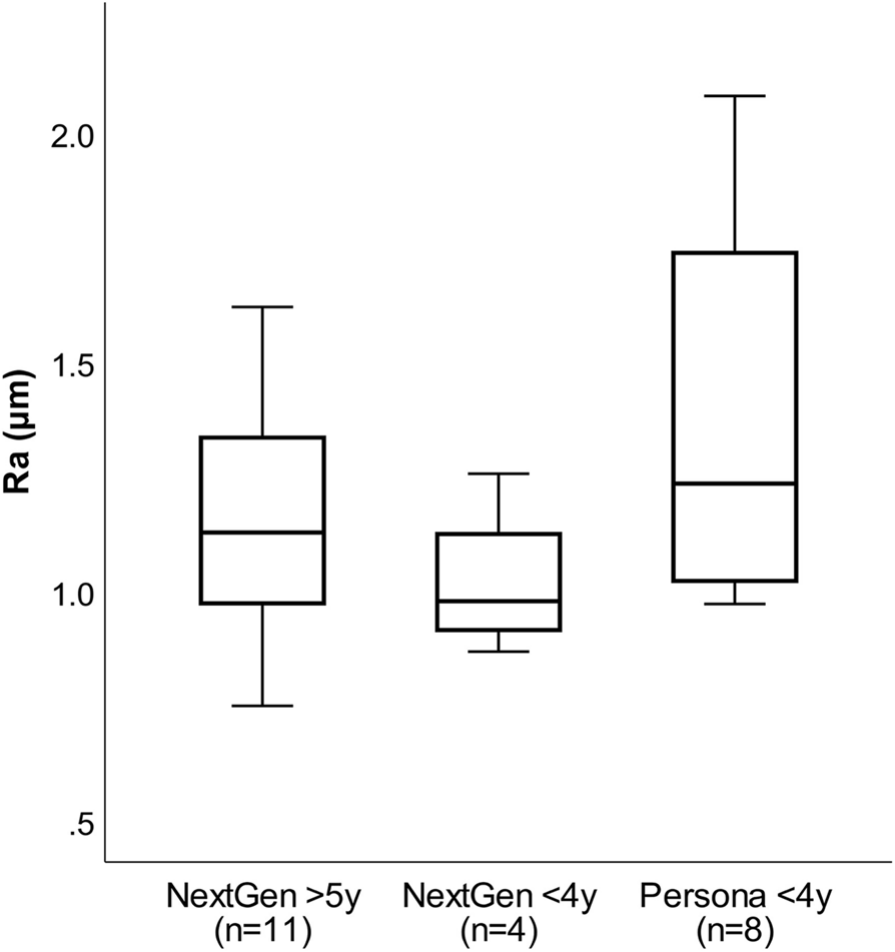
Box plots showing the comparison of backside surface roughness (Ra) of tibial components between the novel anatomic knee system (Persona®) and the predecessor knee system (NexGen®)

A significant difference between Persona® and NexGen® implants was also found for tibial stem roughness values (*p <* 0.05). Persona® stems showed a higher mean surface roughness (1.26 ± 0.44) compared to NexGen® stems (0.89 ± 0.19). Figure 6 illustrates the overall mean roughness values of the tibial trays of the two designs.

### Correlations

Correlations between cement adhesions, implant dimensions, surface roughness, reason for revision and time to revision are demonstrated in Table 4. All calculations have been partialized for type of implants in order to control for time to revision. Hence, it can be stated, that time to revision did not influence the assessed retrieval parameters. There were no significant correlations found between cement adhesions, tibial tray dimensions and surface roughness. Neither were significant correlations described between associated pathologies and the assessed measured values. The few correlations found were either intuitive or random in nature.

**Table 4:** Pearson correlation partialized for type of implant between time to revision, cement adhesion, dimensions and roughness (Ra) of the tibial trays and reason for revision. *** *p <* 0.001, ** *p <* 0.01, * *p <* 0.05

Partial Pearson correlation (corrected for implant type)	Time to revision	Cement adhesion tibial tray	Tibial tray stem length	Tibial tray lip height	Tibial tray thickness with lip	Tibial tray backside, lateral, Ra	Tibial tray backside, medial, Ra	Tibial tray stem, Ra	Total tray backside & stem, Ra	Instability	Malalignment	Patellofemoral	Stiffness	Others
Time to revision	1	−0.22	0.17	−0.32	−0.04	0.16	0.03	−0.12	0.02	0.42	−0.2	−0.29	−0.53*	0.18
Cement adhesion tibial tray	−0.22	1	−0.31	− 0.27	0.12	0.05	0.14	−0.16	0.01	−0.08	0	0.3	0.17	−0.01
Tibial tray stem length	0.17	−0.31	1	0.15	−0.26	−0.07	0.05	−0.01	− 0.01	−0.25	0.33	−0.09	0.06	0.15
Tibial tray lip height	−0.32	−0.27	0.15	1	0.17	0.21	0.2	0.48*	0.32	−0.23	0.18	0.25	0.16	0.01
Tibial tray thickness with lip	−0.04	0.12	−0.26	0.17	1	−0.09	−0.08	− 0.13	−0.11	− 0.03	−0.09	− 0.02	−0.1	0
Tibial tray backside, lateral, Ra	0.16	0.05	−0.07	0.21	−0.09	1	0.88***	0.75***	0.95***	0.33	−0.25	−0.1	− 0.36	−0.18
Tibial tray backside, medial, Ra	0.03	0.14	0.05	0.2	−0.08	0.88***	1	0.7***	0.93***	0.18	−0.25	−0.09	− 0.28	0.03
Tibial tray stem, Ra	−0.12	−0.16	− 0.01	0.48*	− 0.13	0.75***	0.7***	1	0.89***	0.01	−0.04	−0.02	− 0.09	−0.1
Total tray backside & stem, Ra	0.02	0.01	−0.01	0.32	−0.11	0.95***	0.93***	0.89***	1	0.18	−0.2	− 0.08	−0.26	− 0.09
Instability	0.42	−0.08	−0.25	− 0.23	−0.03	0.33	0.18	0.01	0.18	1	−0.39	−0.19	− 0.52*	−0.48*
Malalignment	−0.2	0	0.33	0.18	−0.09	−0.25	− 0.25	−0.04	− 0.2	−0.39	1	0.18	0.16	−0.13
Patellofemoral	−0.29	0.3	−0.09	0.25	−0.02	−0.1	− 0.09	−0.02	− 0.08	−0.19	0.18	1	0.14	−0.06
Stiffness	−0.53*	0.17	0.06	0.16	−0.1	−0.36	− 0.28	−0.09	− 0.26	−0.52*	0.16	0.14	1	−0.17
Others	0.18	−0.01	0.15	0.01	0	−0.18	0.03	−0.1	−0.09	− 0.48*	−0.13	− 0.06	−0.17	1

## Discussion

This retrieval study is the first to examine tibial trays of a novel anatomic knee system and compare them with retrieval findings from its predecessor. Our most important finding was that Persona® tibial tray backsides showed significantly more cement adhesions and higher stem surface roughness values compared to the NexGen® equivalents. While cement adhesions were found on all Persona® tray backsides with a range of 59.4 to 84.7%, there were only cement adhesions found in half of the NexGen® trays (range 32.9 to 51.2%). This supports the content of multiple published research articles dealing with tibial debonding and aseptic loosening across multiple NexGen® tibial components [20, 35, 36]. Hence, one can speculate that there is an issue with the backside of the tibial tray and the cement mantle.

However, these findings cannot be attributed to the material of the implants, as both the novel anatomic and the predecessor knee system were made of the same titanium (Ti-6Al-4 V). Rather, in this context the effect of pre-coating needs to be addressed. TKA coating with a thin layer of polymethylmethacrylate (PMMA) was developed as a means of enhancing the bonding between metal implants and bone cement also made of PMMA [37]. Pre-coating has been mechanically tested against standard surfaces in vitro and found to increase the maximum load to failure at the interface by between 60 and 80% [37]. It had been proposed that pre-coating might be beneficial for long-term implant survival [18, 37, 38]. However, in 2013 Bini et al. evaluated 13′835 pre-coated and 2′713 non-pre-coated primary NexGen® TKAs and did not find favourable results for pre-coated tibial trays with regards to revision rates for aseptic loosening [29]. Regarding our NexGen® cohort, it is a mixed sample consisting of pre-coated and non-pre-coated implants. According to communication with a representative, 81% of NexGen® tibial trays have a PMMA pre-coat with the remaining 19% NexGen® and all Persona® tibial trays not having a pre-coat [36]. With regard to Persona® implants, the literature on debonding and loosening testing is very scarce [39]. A case series reported intraoperative findings of nine patients, amongst them three Persona® implants, who underwent revision TKA due to several reasons [39]. It is striking that the majority of the implants investigated showed femoral and tibial intraoperative debonding and two of the three Persona® implants showed only tibial debonding.

In the course of time, other parameters have also been identified that modify the effect of pre-coating on the strength of the implant–bone interface, such as cement-related factors (cement type and mixing time) as well as surface roughness [18, 29, 40, 41]. These reports have been helpful in identifying specific component design features that may be more prone to debonding, but there is not enough evidence or numbers to prove a certain type of implant has a higher incidence of debonding than any others [39]. In regards to surface roughness, the present study highlights significant differences in backside surface and stem roughness between the different tibial tray designs investigated. Persona® tibial trays and stems showed significantly rougher surfaces compared to NexGen®. This in context with the finding of increased cement adhesions on Persona® tibial trays may indicate that there might be a direct association between surface roughness and cement adhesions. In-vitro test findings of Pittman et al. showed that metal-cement interface strength increases with increasing surface roughness [18]. In particular, samples made of titanium attained stronger bonds with cement when compared with cobalt-chromium (CoCr) ones [11, 18].

Correlation analysis of our data could not find a significant correlation between surface roughness and cement adhesions. The significant difference found in the surface roughness of the tibial tray backside with regards to a rougher surface in Persona® tibial trays seems to be linked to design and coating differences instead of being material-related: both tibial tray designs are made of the same material titanium alloy, therefore the differences might be attributed to the most obvious design alteration with the anatomical tibial tray in the novel knee design as opposed to the symmetrical tray in the predecessor knee system with PMMA pre-coating.

Results from the visual inspection revealed that Persona® stems are significantly shorter with 36.5 mm compared to NexGen® stems (41.2 mm). There is evidence that short stems are associated with an increased rate of aseptic loosening [20, 21, 30]. However, in this study cohort, none of the patients were revised due to loosening. Regarding tibial tray thickness, Persona® implants showed significantly higher values (4.2 mm) in comparison to NexGen® (3.62 mm). Peripheral lips, however, did not show significant differences.

We acknowledge that the surgical implantation technique may affect the cement adhesion; however, Persona® and NexGen® implant procedures were performed by a variety of different high-volume surgeons at different facilities. Given that, it is hard to ascertain specific techniques that may contribute to the amount of cement adhesions; thus, we are unable to further comment on this aspect. However, within the context of cement adhesions, other factors such as cement pockets and viscosity are discussed in literature, too [42–45]. We also acknowledge that one could criticize that the technique of removal may influence the amount of cement left on the tibial component. However, both surgeons used an oscillating saw and chisels during the tibial tray removal.

Similar to all retrieval studies, the present study has a considerable number of limitations [27]. First, it was a small sample size, however, this was the first study of its kind for the Persona® implant. Hence, our results can be used for sample size calculations in future studies with a larger number of retrievals. Second, time to revision was significantly shorter in the Persona® compared to the NexGen® knee system. However, due to this possible influence of time to revision on outcome variables, the differences between implant types were corrected for time to revision for all parameters investigated (Fig. 6). Third, primary TKA was performed by a number of different surgeons, which can cause a wide variety of reasons for failure and thus revision of the implants. However, it is pure speculation whether the reason for revision depends on the surgeon of the primary TKA, the surgical technique, the surgeon indicating revision surgery, the patient or the prosthesis itself. A more homogeneous cohort would certainly benefit the validity of this study. Fourth, we acknowledge that the surgeon itself with his experience and surgical skills may influence the amount of cement left on the tibial component. Fifth, more detailed analysis of demographic data (i.e. BMI and comorbidities) in regards to comparability of the two groups would be of interest and required in future studies. Finally, the evaluation of the clinical benefit remains completely outside the scope of this study. Long-term clinical and longer-term retrieval studies will be necessary to elucidate any clinical advantages of using Persona® implants.

## Conclusions

This is the first comparative retrieval study to investigate cement adhesion and surface roughness on tibial tray backsides as well as its dimensions of a novel knee system, and to compare findings with the predecessor knee design. The comparison of the two designs made from the same material and manufacturer suggested that the novel anatomic knee system showed significantly more cements adhesions and a higher surface roughness which was most likely attributed to the most obvious design and coating alteration of the tibial tray. Future analysis is required to better examine the implant in a larger sample and more multidimensionally to potentially contribute to an improvement in implant design.

## Data Availability

All data generated or analysed during this study are included in this manuscript article. The datasets generated and/or analysed during the current study are not publicly available as they are part of another ongoing research but are available from the corresponding author on reasonable request.

## Abbreviations

UHMWPE: Ultrahigh-molecular-weight polyethylene
PMMA: Polymethylmethacrylate
TKA: Total knee arthroplasty
PE: Polyethylene
PS: Posterior stabilized
CR: Cruciate retaining
MB: Mobile bearing
FB: Fixed bearing
SD: Standard deviation
CoCr: Cobalt chromium

## References

[1] No authors listed. Australian Orthopaedic Association (AOA). National Joint Replacement Registry (NJRR) Hip and Knee Arthroplasty Annual Report 2019. https://aoanjrr.sahmri.com/documents/10180/668596/Hip%2C+Knee%26+Shoulder+Arthroplasty/c287d2a3-22df-a3bb-37a2-91e6c00bfcf0 (accessed Dec 10, 2021).

[2] No authors listed. National Joint Registry for England, Wales, Northern Ireland and the Isle of Man. 16th Annual Report 2019. https://reports.njrcentre.org.uk/Portals/0/PDFdownloads/NJR%2016th%20Annual%20Report%202019.pdf (Accessed 10 Dec 2021).

[3] Hamilton DF, Howie CR, Burnett R, Simpson AH, Patton JT (2015). Dealing with the predicted increase in demand for revision total knee arthroplasty: challenges, risks and opportunities. Bone Joint J.

[4] Abdeen AR, Collen SR, Vince KG (2010). Fifteen-year to 19-year follow-up of the Insall-Burstein-1 total knee arthroplasty. J Arthroplasty.

[5] Piedade SR, Pinaroli A, Servien E, Neyret P (2009). Revision after early aseptic failures in primary total knee arthroplasty. Knee Surg Sports Traumatol Arthrosc.

[6] Weinstein AM, Rome BN, Reichmann WM, Collins JE, Burbine SA, Thornhill TS (2013). Estimating the burden of total knee replacement in the United States. J Bone Joint Surg Am.

[7] Mathis DT, Hirschmann MT (2020). Why do knees after total knee arthroplasty fail in different parts of the world?. J Orthop.

[8] Fehring TK, Odum S, Griffin WL, Mason JB, Nadaud M (2001). Early failures in total knee arthroplasty. Clin Orthop Relat Res.

[9] Sharkey PF, Hozack WJ, Rothman RH, Shastri S, Jacoby SM (2002). Insall award paper. Why are total knee arthroplasties failing today?. Clin Orthop Relat Res.

[10] Mulhall KJ, Ghomrawi HM, Scully S, Callaghan JJ, Saleh KJ (2006). Current etiologies and modes of failure in total knee arthroplasty revision. Clin Orthop Relat Res.

[11] Cerquiglini A, Henckel J, Hothi H, Allen P, Lewis J, Eskelinen A (2019). Analysis of the attune tibial tray backside: a comparative retrieval study. Bone Joint Res.

[12] Rao AR, Engh GA, Collier MB, Lounici S (2002). Tibial interface wear in retrieved total knee components and correlations with modular insert motion. J Bone Joint Surg Am.

[13] Lum ZC, Shieh AK, Dorr LD (2018). Why total knees fail-a modern perspective review. World J Orthop.

[14] Hossain F, Patel S, Haddad FS (2010). Midterm assessment of causes and results of revision total knee arthroplasty. Clin Orthop Relat Res.

[15] Schroer WC, Berend KR, Lombardi AV, Barnes CL, Bolognesi MP, Berend ME (2013). Why are total knees failing today? Etiology of total knee revision in 2010 and 2011. J Arthroplast.

[16] Martin JR, Watts CD, Levy DL, Kim RH (2017). Medial Tibial stress shielding: a limitation of cobalt chromium Tibial baseplates. J Arthroplast.

[17] Martin JR, Watts CD, Levy DL, Miner TM, Springer BD, Kim RH (2017). Tibial tray thickness significantly increases medial Tibial bone resorption in cobalt-chromium Total knee arthroplasty implants. J Arthroplast.

[18] Pittman GT, Peters CL, Hines JL, Bachus KN (2006). Mechanical bond strength of the cement-tibial component interface in total knee arthroplasty. J Arthroplast.

[19] Zhang QH, Cossey A, Tong J (2016). Stress shielding in periprosthetic bone following a total knee replacement: effects of implant material, design and alignment. Med Eng Phys.

[20] Foran JR, Whited BW, Sporer SM (2011). Early aseptic loosening with a precoated low-profile tibial component: a case series. J Arthroplast.

[21] Ries C, Heinichen M, Dietrich F, Jakubowitz E, Sobau C, Heisel C (2013). Short-keeled cemented tibial components show an increased risk for aseptic loosening. Clin Orthop Relat Res.

[22] Hazelwood KJ, O'Rourke M, Stamos VP, McMillan RD, Beigler D, Robb WJ (2015). Case series report: early cement-implant interface fixation failure in total knee replacement. Knee..

[23] Kopinski JE, Aggarwal A, Nunley RM, Barrack RL, Nam D (2016). Failure at the Tibial cement-implant Interface with the use of high-viscosity cement in Total knee arthroplasty. J Arthroplast.

[24] Vanlommel J, Luyckx JP, Labey L, Innocenti B, De Corte R, Bellemans J (2011). Cementing the tibial component in total knee arthroplasty: which technique is the best?. J Arthroplast.

[25] Galea VP, Botros MA, Madanat R, Nielsen CS, Bragdon C (2019). Promising early outcomes of a novel anatomic knee system. Knee Surg Sports Traumatol Arthrosc.

[26] Dai Y, Scuderi GR, Bischoff JE, Bertin K, Tarabichi S, Rajgopal A (2014). Anatomic tibial component design can increase tibial coverage and rotational alignment accuracy: a comparison of six contemporary designs. Knee Surg Sports Traumatol Arthrosc.

[27] Mathis DT, Schmidli J, Hirschmann MT, Henckel J, Hothi H, Hart A (2021). Comparative retrieval analysis of contemporary antioxidant polyethylene: bonding of vitamin-E does not reduce in-vivo surface damage. BMC Musculoskelet Disord.

[28] Zimmer Biomet. NexGen(r) Complete knee solution. Design Rationale. 2004.

[29] Bini SA, Chen Y, Khatod M, Paxton EW (2013). Does pre-coating total knee tibial implants affect the risk of aseptic revision?. Bone Joint J.

[30] Garcia David S, Cortijo Martinez JA, Navarro Bermudez I, Macule F, Hinarejos P, Puig-Verdie L (2014). The geometry of the keel determines the behaviour of the tibial tray against torsional forces in total knee replacement. Rev Esp Cir Ortop Traumatol.

[31] Steere JT, Sobieraj MC, DeFrancesco CJ, Israelite CL, Nelson CL, Kamath AF (2018). Prophylactic Tibial stem fixation in the obese: comparative early results in primary Total knee arthroplasty. Knee Surg Relat Res.

[32] No authors listed. Official J Europ Union Legislation 2021. https://eur-lex.europa.eu/homepage.html?locale=en (accessed 10 Dec 2021).

[33] Cerquiglini A, Henckel J, Hothi HS, Di Laura A, Skinner JA, Hart AJ (2018). Inflammatory cell-induced corrosion in total knee arthroplasty: a retrieval study. J Biomed Mater Res B Appl Biomater.

[34] Di Laura A, Hothi HS, Meswania JM, Whittaker RK, de Villiers D, Zustin J (2017). Clinical relevance of corrosion patterns attributed to inflammatory cell-induced corrosion: a retrieval study. J Biomed Mater Res B Appl Biomater.

[35] Arsoy D, Pagnano MW, Lewallen DG, Hanssen AD, Sierra RJ (2013). Aseptic tibial debonding as a cause of early failure in a modern total knee arthroplasty design. Clin Orthop Relat Res.

[36] Keohane D, Power F, Cullen E, O'Neill A, Masterson E (2020). High rate of tibial debonding and failure in a popular knee replacement: a cause for concern. Knee..

[37] Stone MH, Wilkinson R, Stother IG (1989). Some factors affecting the strength of the cement-metal interface. J Bone Joint Surg (Br).

[38] Davies JP, Singer G, Harris WH (1992). The effect of a thin coating of polymethylmethacrylate on the torsional fatigue strength of the cement-metal interface. J Appl Biomater.

[39] Sadauskas AJ, Engh C, Mehta M, Levine B (2020). Implant interface debonding after total knee arthroplasty: a new cause for concern?. Arthroplast Today.

[40] Davies JP, Harris WH (1993). Strength of cement-metal interfaces in fatigue: comparison of smooth, porous and precoated specimens. Clin Mater.

[41] Shepard MF, Kabo JM, Lieberman JR (2000). The frank Stinchfield award. Influence of cement technique on the interface strength of femoral components. Clin Orthop Relat Res.

[42] Cawley DT, Kelly N, McGarry JP, Shannon FJ (2013). Cementing techniques for the tibial component in primary total knee replacement. Bone & Joint Journal.

[43] Schlegel UJ, Puschel K, Morlock MM, Nagel K (2012). Effect of tibial tray design on cement morphology in total knee arthroplasty. J Orthop Surg res. 2014 Nov 29;9:123. [44] de Uhlenbrock AG, Puschel V, Puschel K, Morlock MM, bishop NE. Influence of time in-situ and implant type on fixation strength of cemented tibial trays - a post mortem retrieval analysis. Clin Biomech.

[44] Kelly MP, Illgen RL, Chen AF, Nam D (2018). Trends in the use of high-viscosity cement in patients undergoing primary Total knee arthroplasty in the United States. J Arthroplast.

[45] Song SJ, Park CH, Liang H, Kang SG, Park JJ, Bae DK (2018). Comparison of clinical results and injury risk of posterior Tibial cortex between attune and press fit condylar sigma knee systems. J Arthroplast.

